# Upper eyelid angiolipoma associated friction in the glide zone

**DOI:** 10.3205/oc000064

**Published:** 2017-05-19

**Authors:** Selman Karaci, Ayten Livaoğlu, Rüştü Köse, Barış Kömür

**Affiliations:** 1 Department of Plastic and Reconstructive Surgery, Kanuni Eĝitim ve Araştirma Hastanesi, Kasüstü, Kaşüstü, Trabzon, Turkey; 2 Department of Pathology, Kanuni Eğitim ve Araştırma Hastanesi, Kaşüstü, Trabzon, Turkey; 3 Department of Plastic Surgery, Recep Tayyip Erdoğan University School of Medicine, Merkez, Rize, Turkey; 4 Department of Ophthalmology, Kanuni Eğitim ve Araştırma Hastanesi, Kaşüstü, Trabzon, Turkey

## Abstract

A 74-year-old man was examined for a mass in the left upper eyelid. It was inconspicuous in inspection. There was no visual restriction, however a feeling of friction on the superior orbit during lid movement. Through eyelid crease lid incision, a solitary lesion appeared in the preaponeurotic space that orginated from the upper tarsal plate. Excisional biopsy revealed adipose proliferation with patch form vessels. This case represents the second reported angiolipoma of the eyelid in English literature.

## Introduction

Angiolipomas are common variants of lipomatous tumors. They most commonly occur on the extremities and trunk of children and adolescents. It is rarely encountered in the orbital region. We present a rare case of angiolipoma within the eyelid with associated friction in the glide zone.

## Case description

**Figure 1:**
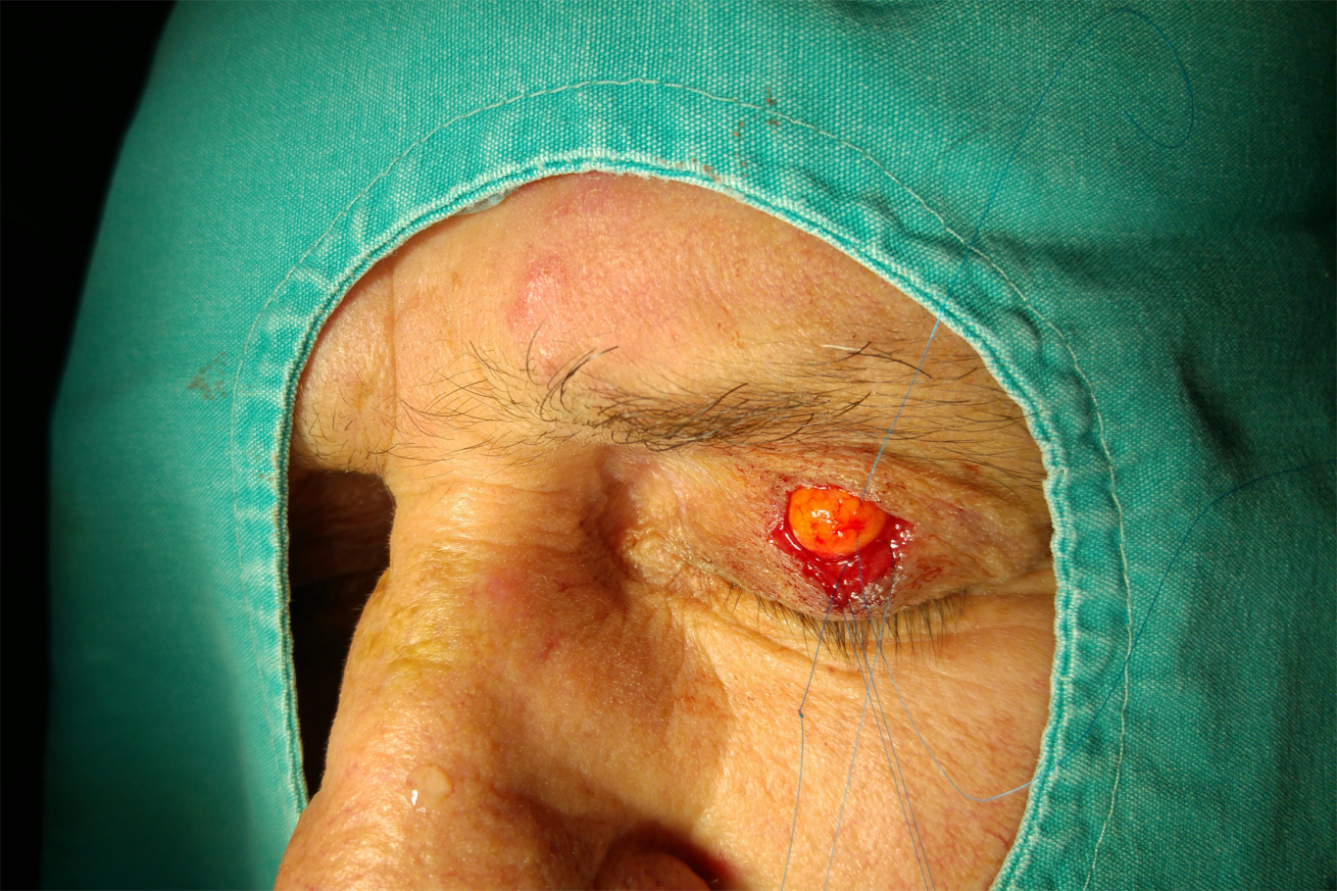
An intraoperative photograph of the lesion showing a mass on the upper eyelid crease, which was adherent to the tarsal plate

**Figure 2:**
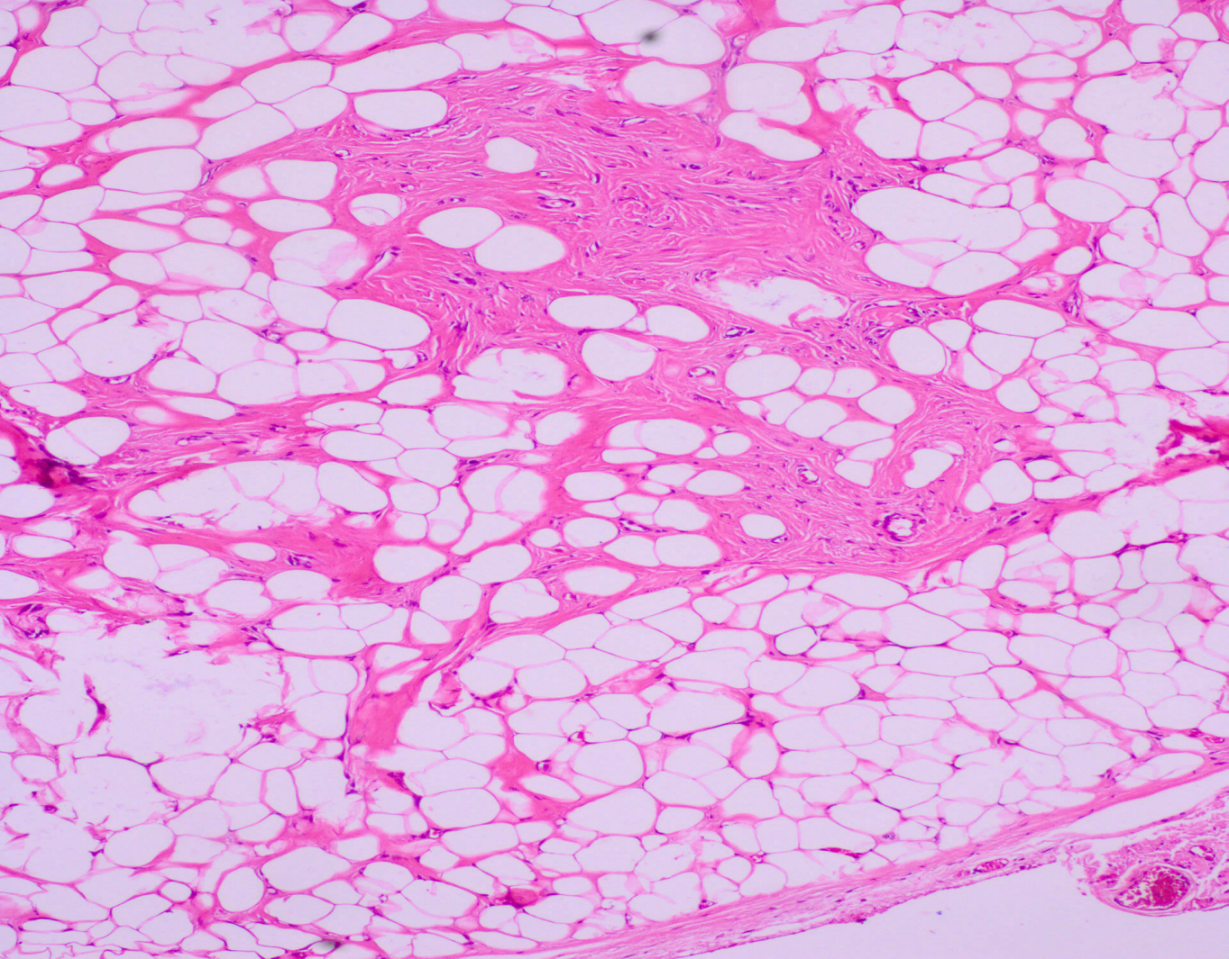
Microphotography of the specimen showing encapsulated tumor mass and mature adipocytes separated by a vascular patch (H & E stain; 10X)

A 74-year-old man was referred to the opthalmology clinic with a history of a mass in the left upper eyelid that had been present for four years. Visual acuity was 7/10 for the right eye and 6/10 for the left eye, anterior segment examination revealed grade 2 nuclear cataract for the right eye and grade 3 nuclear cataract for the left eye. Motility was bilateral normal with no diplopia. Levator functions were bilateral normal, except there was a minimal mechanical ptosis for the left eye. There was also a subcutaneous mass on the left forearm. Systemic check-up relevead no other illness or malignancy. On clinical examination, there was a subcutaneous, firm and round mass located on the central left upper eyelid, which was non-tender to palpation. It was inconspicuous on inspection. There was no eyelid movement restriction but a feeling of friction on the superior orbit during lid movement. Ultrasonography showed a homogeneous echogenic and well defined mass in the anterior orbit. No internal vascularity was seen. An excisional biopsy was taken through the crease of the upper eyelid under local anesthesia. The lesion was 9x7x7 mm in diameter and located 15 mm superior to the eyelid margine and located under the orbicularis muscle. During the removal operation, it was noted that the lesion was stuck to the tarsal border from its base. The mass was removed completely and its location was cauterized while preserving the underlying tarsal plate (Figure 1 (Fig. 1)). Histologically, this nodular lesion, which was composed of mature adipose lobules with a patch of proliferating vascular components and well-circumscribed by fibrous connective tissue, was classified as angiolipoma (Figure 2 (Fig. 2)). Postoperatively, visual acuity was 7/10 for the right eye and 6/10 for the left eye, with no change in the anterior segment examination. The patient did not have any motility restrictions and there was no recurrence during follow-up for the next 12 months. 

## Discussion

Angiolipoma accounts for 6–17% of all lipomas. It rarely occurs in the head and neck region [1]. Eyelid angiolipoma has been reported in only one patient [2] while orbital angiolipoma has been shown in two two cases [3].

Several hypotheses have been proposed for the cause of this lesion: embryonic sequestration of multipotential cells, fatty metamorphosis of a central hemangioma, and vascular proliferation of congenital lipoma. Mast cell-derived vascular endothelial growth factor (VEGF) was suggested as a possible reason for increased vascularity in this particular tumor [1]. 

Angiolipoma characteristically occurs as an encapsulated mass of mature adipose tissue containing clusters of vascular network that was first described by Bowen in 1912. These tumors are frequently tender and occur on the trunk, extremities, and especially forearm of young men. Angiolipomas are classified as infiltrating and non-infiltrating based on the presence of a complete capsule. The infiltrating type is usually poorly encapsulated and has a high recurrence rate after surgical removal [1]. Unlike most adipose tissue tumors, angiolipoma do not have chromosomal alterations. For this reason, it has been proposed that they may represent hamartomas rather than true neoplasm [4].

There have been a few reports of lipomatous variants involving the eyelids: dermolipomas, myolipomas, spindle cell lipomas, and others. The nasopalpebral lipoma-coloboma syndrome associated eye abnormalities and palpebral lipoma are rare congenital abnormalities [5]. 

The upper eyelid consists of two layers; the anterior skin/orbicularis oculi layer and posterior layers of the levator aponeurosis, Müller’s muscle, and the tarsal plate. The glide zone is in the middle of the two layers consisting of the preaponeurotic fat and all potential space. When the levator contracts, the tarsal plate glides up under the skin and orbicularis, therefore the glide zone acts like a friction-free layer [6].

Our patient was experiencing discomfort when looking straight ahead from downgaze. The mass expanded up to the tarsal plate so it was touching the superior orbit. The case presented here is the second reported angiolipoma that appears as an eyelid mass. A histologic variant of lipoma should be considered in diagnosis, if it occurs in unusual locations.

## Notes

### Competing interests

The authors declare that they have no competing interests.
